# The Wreck of All

**Published:** 1869-12

**Authors:** 


### THE WRECK OF ALL.

Many years since, there was a man whom all who knew him respected, loved, revered. His weakly frame, his tottering form, his snowy hair, now lank and long, and a certain subdued expression, such as steady hourly pain seldom fails to impress on the victim of it, aroused the beholder's sympathy; while those who knew him to be engaged for years extending long back into the past, in enterprises of comprehensive good doing, looked upon him as rapidly ripening for heaven. But no one ever noticed, or if so, did not remark upon it, that there was not that majestic mildness in the expression of the face—which conscious integrity and out-gushing lovingness for all the race seldom fail to imprint, nor was there that gladsome twinkle in the eye so fearless of all that is ill. But the absence of these things was readily accounted for in the evident want of bodily health. The most skillful physicians in all the land failed to ferret out the cause of a malady, which seemed to be slowly wearing out its victim. Nineteen years passed away, and the hidden secret was revealed. There was no actual physical disease, no organic destruction going on, but there was a worm remorselessly gnawing at the root of life, eating out its essence in night dreams and in day realities. If ever a smile came on the long wan face, it was mechanical and ghastly; and joyous words and glad expressions fell still-born at the portal of the lips, for the heart never went with them. Those who had known him intimately and long, would not have been greatly surprised on any day, to have heard that he had passed away in his holiness. But what made this wreck of body, what bowed the tall and once manly form, what prematurely bleached the hair to driven snow, and sunk the cheek, and halted the gait, and sapped the foundations of manly strength? It was crime! A crime began a score of years agone, and perpetually renewed for all that weary time, renewed with premeditated intent, with deliberation the most matured, against head, and heart, and conscience. Reason was debased, Religion outraged, and Mercy's cries were stifled, vanquished all, by one pitiless monster, the greed of gold. Against it, for two decades of years, Conscience, all prostrate and powerless, ceased not to wail both day and night together, and in its struggles, failed not to bring its victim to the verge of the grave, and kept him there that dreary while, as if by view of the untried future kept steadily before him, he might be waked up to timely shame and wholesome penitence.

Reader, it is the deathless sting of a wounded conscience, that saps out the life, and by degrees so slow, that it is nothing more nor less than a living martyrdom, lasting so long sometimes, that the casket itself breaks by the strain, just at the time when the jewel, the mind, goes out in madness, and all are wrecked together. To avoid then, one of the most horrible of all deaths, be persuaded to exercise "always a conscience void of offence toward God and toward men."

